# SARS-CoV-2 Seroprevalence in Germany: results from the second wave of the RKI-SOEP study

**DOI:** 10.1093/eurpub/ckac129.047

**Published:** 2022-10-25

**Authors:** E Mercuri, C Poethko-Müller, A Schaffrath Rosario, L Schmid, M Schlaud, A Gößwald, RKI-SOEP-2-study group

**Affiliations:** 1 Department of Epidemiology and Health Monitoring, Robert Koch Institute, Berlin, Germany; 2 Socio-Economic Panel, German Institute for Economic Research, Berlin, Germany; 3 Federal Office for Migration and Refugees, Nurnberg, Germany; 4 Institute for Employment Research, Nurnberg, Germany

## Abstract

**Introduction:**

The first wave of the “Corona Monitoring bundesweit” (RKI-SOEP) study showed that shortly before the start of the German vaccination program only about 2% of adults (> 18 years) had already experienced SARS-CoV-2 infection and more than half of these cases had been detected and notified. The objectives of the second wave of this study are to further investigate the spread of SARS-CoV-2 in Germanýs population aged over 14 years. It aims to determine the seroprevalence of infection- and vaccine-induced IgG antibodies against SARS-CoV-2. Finally, it examines health, demographic and socioeconomic risk and protective factors for infection and vaccine acceptance.

**Methods:**

From November 2021 to February 2022, the second wave of this cross-sectional study collected biospecimens (capillary blood samples) and interview data, including information on infection and vaccination, from a nationwide population sample drawn from the German Socio-Economic Panel (SOEP). The dried self-collected blood samples were then analyzed for the detection of SARS-CoV-2 IgG antibodies by Euroimmun ELISA assay.

**Results:**

Based on preliminary, unweighted data of around 11,000 participants aged >14 years (52% response rate), we expect the final seroprevalence of SARS-CoV-2 antibodies to be in the range of 80 to 90%. Thus, around 10 to 20% of the German population may still be susceptible to a severe disease progression because they are neither infected nor vaccinated. Final results, weighted for non-response and adjusted for test sensitivity and specificity, will be presented.

**Conclusions:**

The RKI-SOEP-2 study will be pivotal in both, contributing to an improved understanding of SARS-CoV-2 propagation in different regional and sub-group settings and in identifying vulnerable target groups that need to be protected against future infections.

**Key messages:**

• Dried blood self-sampling in a nationwide sample is a robust tool to estimate seroprevalence at a population level.

• As of February 2022, presumably 80 to 90% of the German population has previously been infected and/or vaccinated against SARS-CoV-2.

